# Image guidance in radiation therapy for better cure of cancer

**DOI:** 10.1002/1878-0261.12751

**Published:** 2020-06-29

**Authors:** Vincent Grégoire, Matthias Guckenberger, Karin Haustermans, Jan J. W. Lagendijk, Cynthia Ménard, Richard Pötter, Ben J. Slotman, Kari Tanderup, Daniela Thorwarth, Marcel van Herk, Daniel Zips

**Affiliations:** ^1^ Department of Radiation Oncology Léon Bérard Cancer Center Lyon France; ^2^ Department for Radiation Oncology University Hospital Zurich University of Zurich Switzerland; ^3^ Department of Radiation Oncology Leuven Cancer Institute University Hospital Gasthuisberg Leuven Belgium; ^4^ Department of Radiotherapy University Medical Center Utrecht The Netherlands; ^5^ Centre Hospitalier de l'Université de Montréal Canada; ^6^ Department of Radiation Oncology Medical University General Hospital of Vienna Austria; ^7^ Department of Radiation Oncology Amsterdam University Medical Centers The Netherlands; ^8^ Department of Oncology Aarhus University Hospital Denmark; ^9^ Section for Biomedical Physics Department of Radiation Oncology University of Tübingen Germany; ^10^ Department of Biomedical Engineering and Physics Cancer Center Amsterdam Amsterdam UMC University of Amsterdam The Netherlands; ^11^ Institute of Cancer Sciences University of Manchester UK; ^12^ Department of Radiotherapy Related Research The Christie NHS Foundation Trust Manchester UK; ^13^ Department of Radiation Oncology University of Tübingen Germany

**Keywords:** adaptive radiotherapy, brachytherapy, cone‐beam CT, image guidance, molecular imaging, MR‐linac, radiation, stereotactic radiotherapy

## Abstract

The key goal and main challenge of radiation therapy is the elimination of tumors without any concurring damages of the surrounding healthy tissues and organs. Radiation doses required to achieve sufficient cancer‐cell kill exceed in most clinical situations the dose that can be tolerated by the healthy tissues, especially when large parts of the affected organ are irradiated. High‐precision radiation oncology aims at optimizing tumor coverage, while sparing normal tissues. Medical imaging during the preparation phase, as well as in the treatment room for localization of the tumor and directing the beam, referred to as image‐guided radiotherapy (IGRT), is the cornerstone of precision radiation oncology. Sophisticated high‐resolution real‐time IGRT using X‐rays, computer tomography, magnetic resonance imaging, or ultrasound, enables delivery of high radiation doses to tumors without significant damage of healthy organs. IGRT is the most convincing success story of radiation oncology over the last decades, and it remains a major driving force of innovation, contributing to the development of personalized oncology, for example, through the use of real‐time imaging biomarkers for individualized dose delivery.

## Graphical abstract

Sophisticated, high‐resolution, real‐time image‐guided radiotherapy (IGRT) using X‐rays, computer tomography, magnetic resonance imaging, or ultrasound, enables delivery of high radiation doses to tumors without significant damage of healthy organs. Here, we review IGRT research and applications and discuss how they contribute to the development of personalized oncology, for example, through the use of real‐time imaging biomarkers for individualized dose delivery.

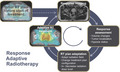

**Abbreviations**
ART: response‐adaptive radiotherapy
CBCT: cone‐beam CT
CT: computer tomography
CTV: clinical target volume
GTV: gross tumor volume
HNSCC: head‐and‐neck squamous cell carcinoma
IGRT: image‐guided radiotherapy
IMRT: intensity‐modulated radiation therapy
linac: linear accelerator
MRI: magnetic resonance imaging
NKI: Netherland Cancer Institute
NSCLC: non‐small‐cell lung cancer
OAR: organs at risk
OMD: oligometastatic disease
PET: positron emission tomography
PTV: planning target volume
RT: radiotherapy
SABR: stereotactic ablative radiotherapy
SBRT: stereotactic body radiotherapy
SMART: stereotactic MR‐guided adaptive radiation therapy
TRUS: trans‐rectal ultrasound
VMAT: volumetric‐modulated arc therapy

## 1 Introduction

Radiation therapy aims for destroying the tumor without damaging the surrounding normal tissues and organs. Radiation doses required to achieve sufficient cell kill in cancers exceed in most clinical situations the dose that is tolerated by the normal tissues, especially when large parts of the respective organ are being irradiated. This delicate balance between the radiation dose–response relationship for tumor cell kill and probability of normal tissue toxicity represents the core principle and also the main challenge of radiation oncology. Coverage of the tumor and sparing of normal tissues is the main optimization approach of high‐precision radiation oncology. In the late 19th century, X‐ray radiation was limited in energy and, therefore, radiation therapy was limited to superficial neoplasms such as of the skin. By contrast, high‐energy radiation beams used nowadays target with geometric precision of millimeters virtually all tumors in the body including brain, lung, breast, prostate, etc. For the treatment of superficial tumors, the radiation beam can be adjusted by eye to ensure full coverage of the tumor and sparing of critical adjacent organs. However, medical imaging is required for the precise adjustment of radiation beams targeting tumors that are located in the inner of the body of the patient. Medical imaging for tumor localization during the preparation phase, as well as in the treatment room for localization of the tumor and directing the beam is referred to as image‐guided radiotherapy (IGRT). Sophisticated high‐resolution and real‐time IGRT using X‐rays, computer tomography (CT), magnetic resonance imaging (MRI), or ultrasound constitutes the basis of modern radiation oncology, enabling the delivery of high radiation doses to tumors without significant damage of healthy organs. IGRT is the most convincing success story of radiation oncology over the last decades. And it remains a major driving force of innovation, contributing to personalized oncology through the development and implementation of real‐time imaging biomarkers for individualized and real‐time control of dose delivery. In this review article, we describe current developments in IGRT and comment on avenues for further research. CT‐based IGRT, high‐precision image‐guided stereotactic ablative radiation oncology, image‐guided brachytherapy in gynecological and prostate cancers, molecular imaging with positron emission tomography (PET), and 0.35T hybrid‐ and high‐field MR‐linear accelerator (linac) are discussed. In addition, we review quantitative imaging for response‐adaptive radiation oncology.

## 2 CT‐based IGRT

Up to the beginning of this century, most image guidance was based on 2D imaging, that is, MV imaging using the treatment beam in combination with a specialized image detector [1] or kV imaging using one or two independent kV sources and standard X‐ray image detector [2]. As 2D imaging only allows identification of rigid and radio‐opaque objects (bones, implanted markers), there is an obvious benefit of integrating 3D imaging with the treatment machine, such that the tumor and surrounding organs at risk (OAR) can be localized prior to each treatment fraction. This allows the treatment then to be adapted to compensate for changes in the absolute and relative position of target and surrounding OAR. Even though acquisition of 3D images is somewhat slower, its interpretation is easier, faster, and more accurate than of planar imaging, and therefore, volumetric imaging has become the de facto standard of image guidance in current radiotherapy (RT). In RT, as well as in surgery, standard diagnostic CTs were first utilized for guidance, either placed on rails or with robotic movement of the patient from the treatment position into the imaging position. Later, cone‐beam CT (CBCT) was used, which features a more compact design. This allows CBCT to be integrated with the gantry of a treatment machine, or be placed on an independent robot. This section presents a roughly chronological overview of these technologies, listing some of their advantages and disadvantages, and describing some clinical applications.

The first reported integration of diagnostic CT systems in the RT treatment room was reported in New York and Houston [3]. The advantage of these systems is that they provide diagnostic image quality, but they have the major disadvantage that the CT scanner cannot share the same isocenter as the treatment machines. This means that one must move the patient between devices, and correct for mechanical instability and patient motion in the transition, or only perform relative localization, with the absolute localization provided by other (e.g., planar) imaging on the treatment machine. The advantage of using a readily available CT device is also a disadvantage, because they suffer from a lack of integrated software for image guidance, making workflows less efficient.

Some of the clinical applications of in‐room CT include studies on head‐and‐neck deformation in Houston [4], applications in proton and particle therapy [5], and integration with the Cyberknife [6].

Modern CT developments such as dual‐energy CT can be readily integrated in the in‐room CT approach with this approach, making it an attractive solution for dose calculation in particle therapy.

Tomotherapy was proposed by Mackie *et al*. in 1992 [7]. It involves mounting a linac on a CT scanner like gantry, using so that the same radiation source for treatment and imaging. The system utilizes the unique properties of a gridded xenon filled detector to achieve a very high quantum efficiency at the used high photon energies for imaging, reducing soft tissue contrast. High energies are required because the 6 MV treatment source cannot go down to diagnostic energies but operates around 300 kV. The obvious advantage of tomotherapy is that it requires only a single source. As imaging at high energy is insensitive to metal artifacts, tomotherapy is ideally suited to treat patients with metal implants. A major disadvantage is that the detector is single slice, therefore, the amount of time required to scan a region of interest is directly proportional to its length with an imaging time of about 6 s per slice. Clinical application of tomotherapy is quite broad, ranging from prostate to head and neck RT. Recent developments include the addition of tumor tracking to the device [8].

**Fig. 1:**
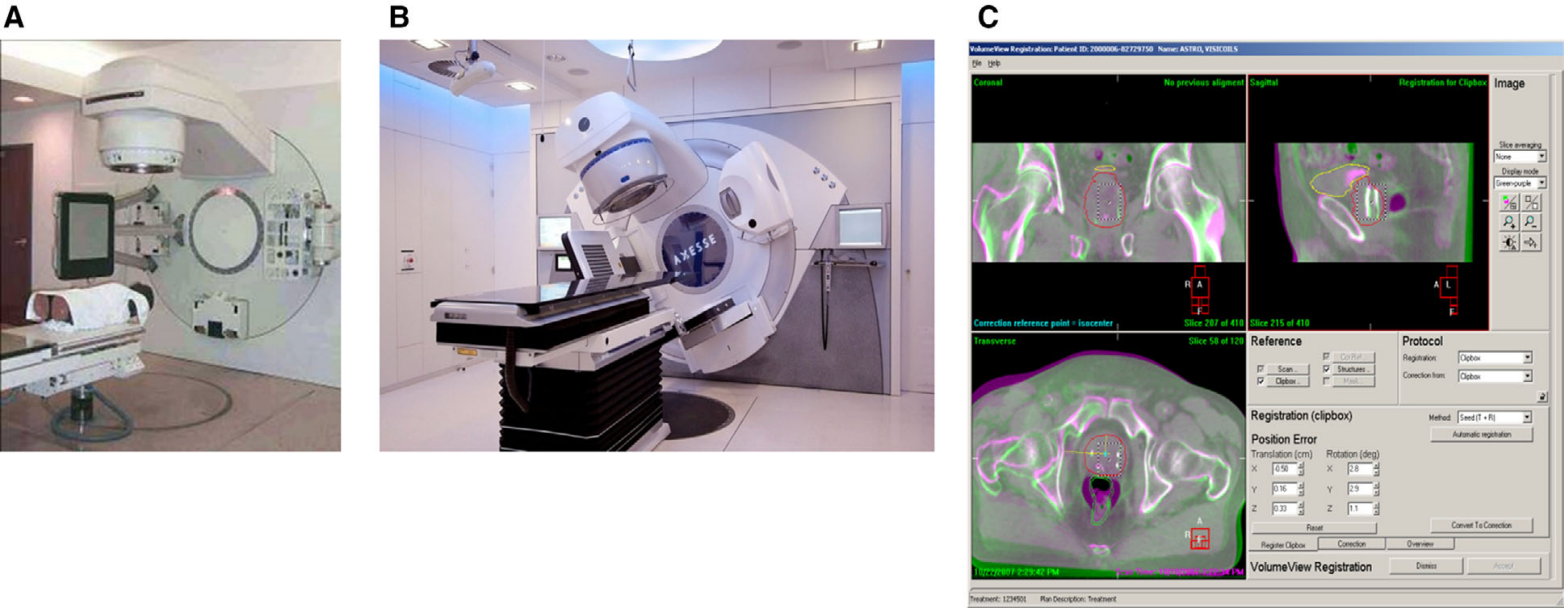
(A) Initial CBCT prototype by Jaffray *et al*. (B) Modern integrated CBCT system. (C) Software system for image reconstruction and analysis illustrating a workflow‐based design.

Integration of CBCT on an accelerator (gantry‐based CBCT) was first proposed by Jaffray and Wong around 1997; the first prototypes were constructed in Beaumont and Toronto prior to the year 2000 in collaboration with Elekta Fig. 1 [9]. The early development of high‐speed 3D and 4D image reconstruction software, as well as practical workflow software at the Netherland Cancer Institute (NKI), allowed the system to be put into clinical use quite early [10]. NKI commenced routine clinical use with in‐house software in 2004 [11]. The first product was released by Elekta in 2005, with software developed by NKI, with Varian following a few years later.

The main advantage of CBCT‐IGRT is its integration with the treatment machine, providing a calibrated isocenter position (even though the isocenter coincidence must verified regularly [12]. This means that no patient or table motion is required between imaging and treatment. Current CBCT‐IGRT systems provide integrated software solutions focusing on localization of tumor and/or OAR.

Since 2009, 4D CBCT imaging is available commercially facilitating visualization of tumors that move under respiratory motion without motion blurring. This is a requirement for accurate image guidance of mobile tumors moving over 1 cm pp [13]. Since 2012, CBCT imaging during volumetric arc therapy (VMAT) delivery is available [14], providing an efficient method of verification imaging during hypofractionated delivery, that is, to check whether the planned tumor position is actually achieved during therapy. Disadvantages of CBCT are (a) that image acquisition is slow on an open gantry due to legal gantry speed limitation (International Electrotechnical Commission limit is 1RPM), (b) the image quality of CBCT is somewhat poorer than fan‐beam CT due to the large amount of X‐ray scatter emanating from the patient (which is software corrected or rejected by a scatter grid). But the main limitation of CBCT is a poor image quality in regions of the body with much internal motion due to respiration and gas, causing blurring of soft tissue interfaces.

Currently, CBCT‐IGRT is the most common form of image guidance used in the clinic; millions of patients have been treated with such systems. CBCT‐IGRT allows shrinking of safety margin, for example, reducing rectal toxicity in the treatment of prostate cancer and enabling frameless radiosurgery of brain and lung tumors. Integrated with proton therapy machines, CBCT is particularly well suited for treatment of tumors in the brain, head and neck, lung, breast, or extremities. CBCT is very well suited for tumors in the brain, head and neck, lung, breast, and extremities, but less so for abdominal organs due to motion. In combination with breath‐hold, CBCT guidance for abdominal organs is feasible.

The efficacy of CBCT‐IGRT is expected to increase further through better reconstruction algorithms (iterative reconstruction now available in Varian software). Integration of efficient scatter grids [15], integration on ring‐based gantries with very good mechanical stability and faster rotation [16]. Faster and lower dose image acquisition (image gently) especially for pediatric patients receiving proton therapy [17].

Robotic CBCT place the kV source and detector on a robotic device that can be attached to the ceiling [18] or to the patient table [19]. These systems are mainly utilized for particle therapy because they allow scanning motion independent of gantry rotation (which can be slow in particle therapy) and for RT systems that lacks a gantry. Advantages are that these are universal systems that can be used in multiple disciplines. Robotic CBCT systems allow faster scanning than open gantry systems due to their smaller dimensions. Also robotic systems allow complex scanning geometries (offset and helical) enabling larger fields of view. Disadvantages of robotic systems are cost, the potential for collision, and the increased amount of scatter due to smaller distances of patient to detector.

Overall, CT‐based guidance is the current standard for image guidance. It is very well suited for treatment of tumors in the brain, head and neck, lung, breast, or extremities, but less so for abdominal organs due to motion blurring (CBCT) or motion distortion (CT). Often CT imaging dose is mentioned as a major disadvantage, but the use of acquisition protocols that are consistent with the image guidance task, the imaging dose can be reduced compared to diagnostic tasks and tends to be insignificant compared to the treatment dose, scatter, and leakage. The increased accuracy of image guidance, however, requires further optimization of all RT processes [20].

## 3 MR‐based IGRT

### 3.1 The 0.35T hybrid MR‐linac

In 2012, ViewRay (ViewRay Inc., Cleveland, OH, USA) introduced an integrated magnetic resonance‐guided RT system (MRIdian). This system combined a 0.35T MRI with a robotic three‐headed ^60^Co RT system [21]. In 2017, the RT part of the system was replaced by a 6 MV linac.

**Fig. 2:**
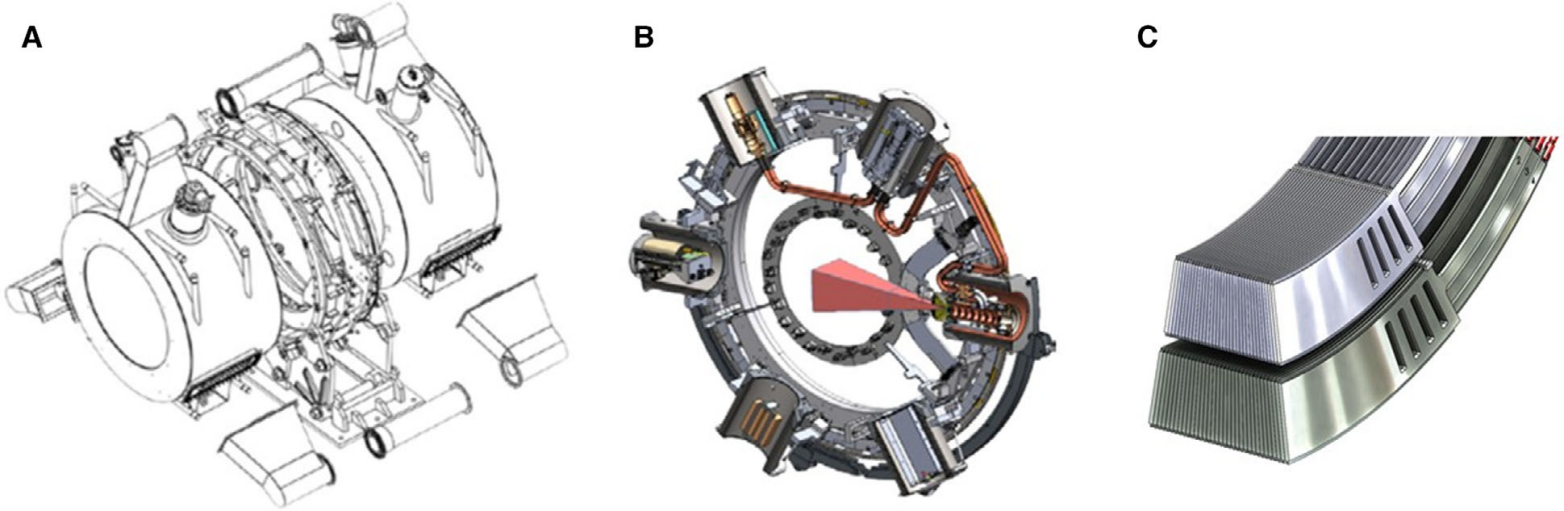
ViewRay system consists of 0.35T split magnet MRI system (A), with a rotating gantry housing a 6 MV linac (B), equipped with 138‐leaf double‐focused double‐stacked multileaf collimator (C).

The MRIdian linac system uses a split magnet MRI system, combined with a rotating gantry that houses the linac Fig. 2. The double‐focused and double‐stacked multileaf collimator leads to a very sharp beam penumbra, making it optimally suited for stereotactic treatments as well. In addition, the system comes with a software system that enables the immediate and rapid use of the actual anatomical imaging into an adapted treatment plan. MR imaging during the beam delivery allows for additional control and gated delivery. In January 2014, the first patient was treated on the MRidian Cobalt system at Washington University, St. Louis, and later that year, the first adaptive treatments were delivered [22]. A small number of ^60^Co systems have been installed, and currently, around 20 MRIdian linac systems are operational worldwide.

The use of MRI guidance on the linac offers various benefits. First of all, the improved soft tissue imaging over CBCT scanning obviates the need for implanted markers. A clear view of the tumor and surrounding organs risk makes it possible to use smaller safety margins. Secondly, the ViewRay software makes it possible to adapt the treatment plan to the anatomy of the day. This avoids high doses to critical structures in the vicinity of the tumor and an improved coverage of the tumor. The third major benefit is that for the first time in the history of RT, the tumor and organs around it can be visualized continuously during the delivery of the beam. This enables a more precise tracking of the delivered dose, but also the ability to shut down the beam immediately if unfavorable conditions occur during the treatment (e.g., passing of rectal gas during irradiation for prostate cancer). The group at VUmc in Amsterdam developed a system that enables the patient to see the MR images with indicated tumor during treatment and use it for the breath‐hold gated delivery [23]. These benefits are anticipated to result in improved tumor control and reduced side effects. The time per treatment fraction is significantly longer compared to conventional treatments, especially when daily adaptation is performed [24]. Therefore, daily treatment plan adaptation is often reserved for hypofractionated treatments. Daily plan adaptation leads to a different workflow and involvement of radiation oncologists and medical physicists with every treatment session. Some centers have dedicated radiation oncologist and physicist continuously present at the machine.

Although the MRIdian system can be used for virtually all indications, stereotactic MR‐guided adaptive radiation therapy (SMART) has primarily been used for moving tumors in thorax and upper abdomen and in the pelvis. To reduce the time needed for contouring, an approach has been developed where only the OAR in the region in the first centimeters around the tumor are recontoured [25].

In pancreatic cancer, SMART leads to improved target coverage and better sparing of OAR [26,27]. An evaluation of 180 adaptive treatment fractions showed that the percentage of plan meeting all dose constraints increased from 44% to 83% [26]. The benefit of daily plan adaptation was observed in about half of the fractions and mainly when the distance between tumor and OAR was 3 mm or less [26]. A nonrandomized comparison of patients with inoperable pancreatic tumors treated with SMART to higher compared to standard doses showed improved local control and improved overall survival [28], indicating that this approach holds many promises.

In patients with central [29] and ultracentral [30] lung tumors, at increased risk for toxicity, plan adaptation resulted in fewer violations of treatment planning constraints. In over 90% of fractions, the optimized treatment plan was chosen and the coverage of the tumor was improved, while excessive doses to surrounding structures where avoided. This widens the therapeutic window of stereotactic treatments for this group of lung cancer patients.

There are many other indications where SMART has already shown to be of benefit. Examples are liver tumors, adrenal metastases, kidney tumors, and prostate tumors. To generate clinical evidence, a number of prospective studies have been initiated.

### 3.2 High‐field MR‐linac

**Fig. 3:**
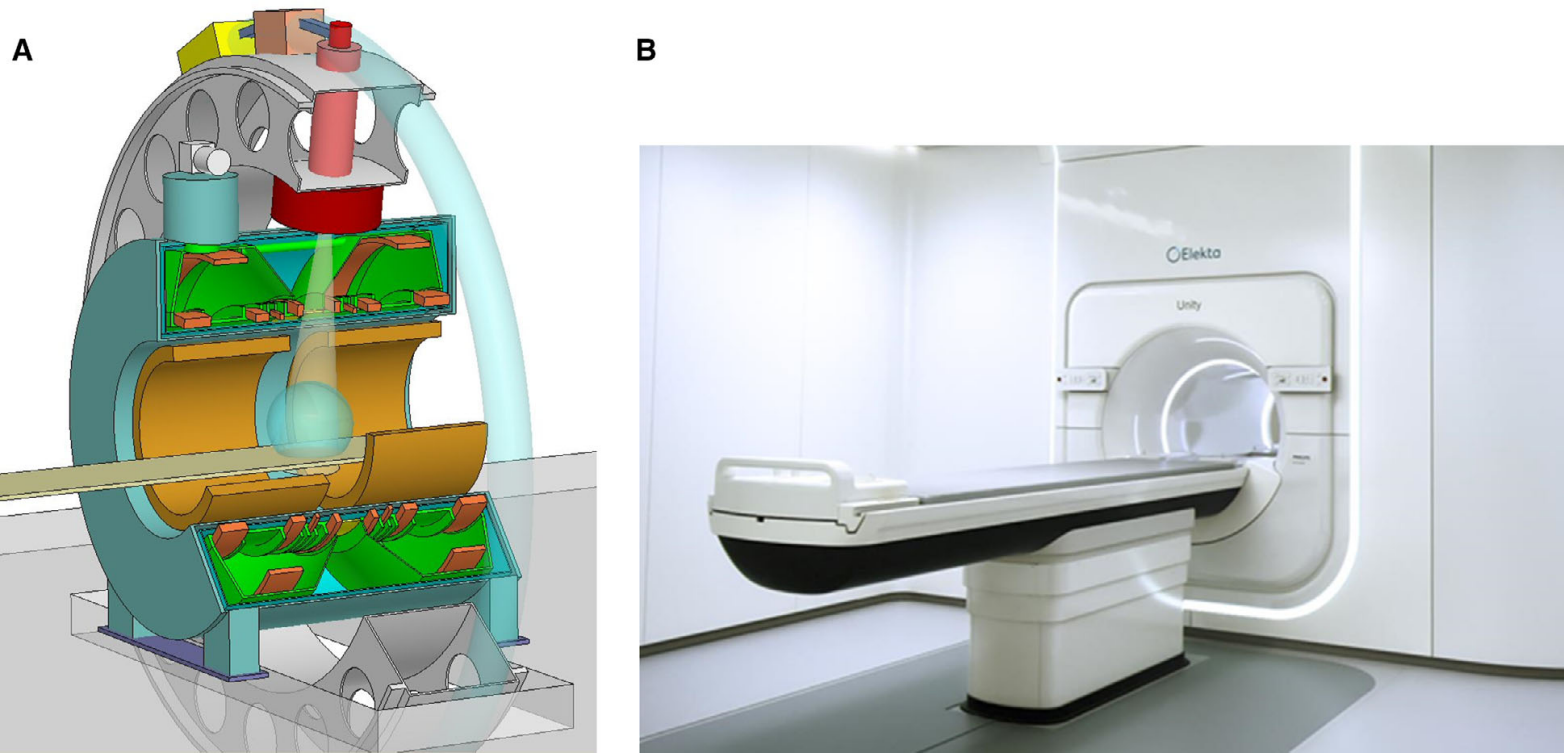
(A) Schematic design and (B) Elekta Unity system.

Using active magnetic shielding the magnetic field just outside an MRI can be minimized, allowing the positioning of a linac gantry in this zone, fully decoupling the two systems Fig. 3. This makes it possible to combine a high‐field MRI with a regular RT accelerator, with the accelerator rotating around the MRI cryostat. Such a system is capable to deliver diagnostic quality 1.5T MR images, while the accelerator dynamically delivers its dose [31,32]. The radiation beam has to pass through the MRI cryostat. This results in some beam attenuation and an isocenter source distance of about 1.45 m. Within the cryostat, a radiation transparent window has to be created without superconducting wires. To let the beam pass also the gradient coil has been split. This window limits the caudal cranial field size to about 22 cm. Wider gaps will reduce the image quality of the MRI. Using this design, the Elekta Unity can execute diagnostic examcards designed for Philips 1.5 T MR radiology systems, while having stereotactic precision RT dose delivery [33]. The Unity system is by its diagnostic quality 1.5 T MRI prepared for functional imaging during treatment delivery.

Dose deposition is thus performed with the patient inside the 1.5 T magnetic field. The magnetic field changes the dose deposition by influencing the tracks of the secondary electrons. Those electrons take curved paths, resulting in a slightly shifted beam profile and the electron return effect at tissue–air interfaces [34]. These phenomena are well investigated and relatively easy to handle, but requires that the dose engine of the treatment planning system has been based on Monte Carlo code. This is to assure that the physics are well taken into account.

The online MR images allow treatment optimization at a daily base. The online MRI is being registered to the simulation CT to provide the Hounsfield patient data. The online image is being registered to the simulation MRI to obtain the tumor and OAR contours, contours are adjusted if needed. With this information, a new treatment plan is being created [35]. This online adaptive procedure not only deals with translations, but also with rotations, deformations, and tumor regression, making table translations and rotations obsolete.

The MRI offers the possibility to visualize the real‐time 3D anatomy of the patient at the exact moment of irradiation. Making the MRI that fast that anatomy can be followed on a subsecond time scale is currently investigated in several institutes. It will be clear that this real‐time visualization also allows real‐time dose accumulation and thus real‐time dose optimization. The blue‐sky will be that a patient anatomy is being followed in real time, while the dose delivery is being optimized continuously, making the treatments robotic self‐navigating optimizations.

Online MRI also allows that the treatment procedure may become an interventional one. MRI‐guided modifications of the anatomy for OAR sparing, like temporarily spacers between rectum wall and prostate, must be evaluated.

To generate clinical evidence an international consortium was founded, the R‐IDEAL framework was developed and all groups are collaborating in the multiple outcome evaluation of RT (Momentum) database [36]. The objective of this database is to generate clinical and imaging data to evaluate treatment outcome and to act as a repository for evaluation and training. Clinical trials currently explore the benefits of MR‐guided RT in patients with cancers of the liver, esophagus, bladder, brain, lung, rectum, head and neck, prostate and breast as well as in oligometastatic disease (OMD) [37, 38, 39, 40, 41, 42, 43, 44, 45, 46].

High‐field MR‐linacs have the potential to become the next‐generation RT standard. The concept of seeing what you treat is extremely strong. Such a development would transform RT toward an interventional radiology procedure, making wide imaging knowledge a prerequisite. Stereotactic precision dose painting according to actual tumor presence, tumor characteristics, and OAR sensitivity will become the new standard, driving RT toward concentration in broader and larger departments.

## 4 Molecular imaging with positron emission tomography in radiation oncology

With the routine use of intensity‐modulated radiation therapy (IMRT) or VMAT allowing highly conformed dose distributions, there is an increasing need for refining the delineation of the gross tumor volumes (GTV). Molecular imaging, also known as biological imaging or functional imaging, is the use of noninvasive imaging techniques that enable the visualization of various biological pathways and physiological characteristics of tumors and/or normal tissues. It mainly refers (but is not limited to) PET, which, with the use of various tracers, offers the opportunity to improve diagnostic accuracy and to integrate tumor biology mainly related to the assessment of tumor cell density, tumor hypoxia, and tumor proliferation into the treatment planning process [47]. Furthermore, with the apprehension of the heterogeneity in tumor biology with molecular imaging, growing evidence has been collected over the years to support the concept of dose escalation/dose redistribution using a planned heterogeneous dose prescription, the so‐called ‘dose painting’ approach. Validation trials are ongoing, and in the coming years, we expect to position the dose painting approach in the armamentarium for the treatment of patients.

In the following PET image acquisition, reconstruction and segmentation with special consideration for radiation oncology are discussed. The usage of PET for target volume selection and delineation has much stronger requirements in terms of image quality in comparison with diagnostic PET imaging. PET has a rather low spatial resolution in the order of 5 mm, and a high level of noise due to the rather low number of emitted and detected photons as a consequence of the limited activity that can be administered to patients for obvious radioprotective reasons. To circumvent these limitations, PET images are typically acquired in 3D mode, are corrected for scatter, attenuation, random events, and dead time, and, if available, acquired using time‐of‐flight measurements, or new crystal scintillators and silicon photomultipliers to improve both time and space resolution [48]. PET image reconstruction is routinely done using iterative algorithms, and postreconstruction processing such as the use of denoising, deblurring, or edge‐preserving filters can be used to further enhance image quality [49]. Accurate delineation of the tumor volume and shape from PET images remains an open challenge. Different delineation methods, validated for specific tumor sites, and to various extents, on phantoms, synthetic images, other imaging modalities like CT, or ground truth have been proposed [50].

Clinical evidence for the use of PET for target volume delineation has been demonstrated for FDG‐PET mainly in head and neck and in lung cancer. In locally advanced head and neck squamous cell carcinoma (HNSCC), FDG‐PET‐based GTV definition has been shown to be more accurately related to the macroscopic tumor specimen. Its use for planning purposes was associated with a significant reduction of the clinical target volume (CTV) and planning target volume (PTV), as well as with a sparing of critical normal tissues when IMRT treatment was used [51]. In non‐small‐cell lung cancer (NSCLC), the use of FDG‐PET has been shown to change the delineation of the primary tumor GTV by discriminating tumor tissue from atelectasis or necrosis and to improve the delineation of positive mediastinal lymph nodes [52]. Such volume modifications also translated into modification of the dose distribution. Methodological issues related to breathing motion have been identified and four‐dimensional PET acquisition has been shown to improve tumor visualization and accuracy of volume reconstruction [53]. In esophageal carcinoma, no definite data support the use of FDG‐PET for the primary tumor delineation, but it could have benefit in individualizing positive lymph nodes outside of the mediastinum [54]. In cervix carcinoma, FDG‐PET has been shown to be very specific for the selection and delineation of para‐aortic lymph nodes, but has not shown any benefit for the primary tumor delineation [55]. In the brain, owing the high physiological FDG uptake, other PET tracers have been studied, and ^11^C‐methionine has been shown to have an added value in delineating recurrent tumor, glioma, or meningioma [54]. Last in prostate carcinoma, ^68^gallium‐ or ^18^F‐PSMA has shown promising results for the management of recurrent disease and ongoing studies indicate that these tracers may outperform conventional imaging [56].

**Fig. 4:**
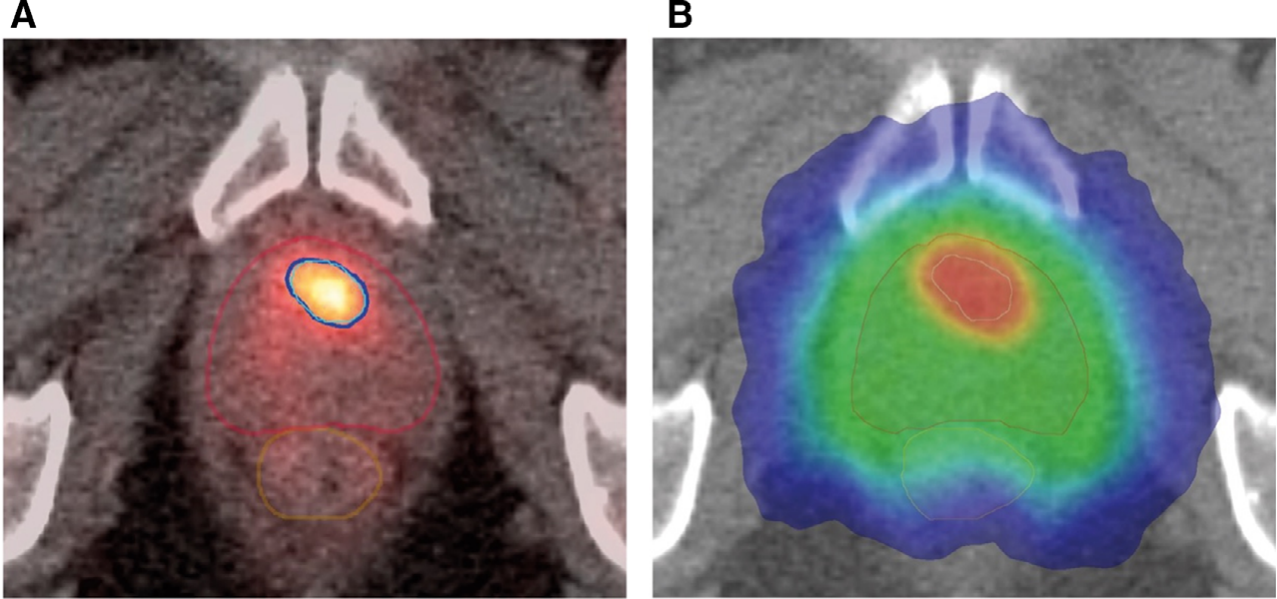
PSMA‐PET‐based focal boosting in prostate cancer. (A) Axial PSMA‐PET‐CT slice showing the contours of the prostate (red), GTV (cyan), rectum (brown), and the 50 Gy isodose (5 fractions of 10 Gy; marine blue). (B) Corresponding CT slice with color wash isodose curve showing conformal dose shaping to the prostate (clinical tumor volume) treated to 35 Gy in five fractions of 7 Gy and intraprostatic tumor (GTV) with sparing of the rectum and urethra. The intraprostatic lesion (cT1c, Gleason 3 + 4 = 7, iPSA = 16.6 ng·mL^−1^) is located in the left transition zone. The patient participated into the multicenter prospective phase II hypo‐FLAME study (NCT02853110, ClinicalTrials.gov).

Radiation dose painting, that is, the prescription and delivery of a nonuniform dose to the CTV, is a different paradigm in radiation therapy [57]. The basic idea is to replace, completely or in part, the morphologically, or anatomically defined target volumes with a map of the spatial distribution of a specific tumor phenotype that is hypothesized or has been shown to be related to local tumor control after RT Fig. 4. A dose prescription function is then used to transform this map into a map of prescribed doses that can be used as input to an inverse planning optimizer, either to increase or to redistribute the prescribed dose.

The current interest in dose painting focuses mainly on three evidence‐based causes of RT failure in the clinic: tumor burden or tumor cell density, tumor cell proliferation, and tumor hypoxia. For tumor burden, a prospective randomized phase II dose painting and dose escalation study was recently reported in locally advanced HNSCC, whereby the prescribed dose was increased up to 81 Gy on the FDG‐PET avid area. This study showed a statistically significant improvement in local control (from 75% after 69 Gy to 88% after dose painting at 2 years) without long‐term mucosal ulceration providing that the 80‐Gy isodose volume does not exceed 1.75 cm^3^ [58]. Tumor hypoxia, which can be imaged using various ^18^F‐labeled PET tracers, has been observed in a large variety of human tumors, and its presence was correlated to local relapse after RT in head and neck carcinoma [59]. In proof‐of‐concept planning studies, it has been calculated that a dose increase to the tumor hypoxic area by 15–20% could substantially increase the control probability without affecting normal tissue toxicity (see review in [51]. Conversely, it has also been shown that in patients with HPV‐positive oropharyngeal carcinoma, a reduction of the prescribed radiation dose of 10 Gy to the lymph nodes in patients showing a resolution of hypoxia after 1 week of treatment was a safe approach [60]. Regarding cell proliferation, the fluorinated thymidine analog ^18^FLT has been used in human tumors to define subtarget volumes that might get an additional radiation dose level (see review in [51]. However, in the absence of direct clinical evidence for an association between these regions and a subsequent local treatment failure, the biological rationale for this boost strategy is still not completely clear, and further data are thus needed before using dose painting based on pretreatment FLT distribution.

## 5 Image‐guided high‐precision stereotactic ablative radiation oncology

**Fig. 5:**
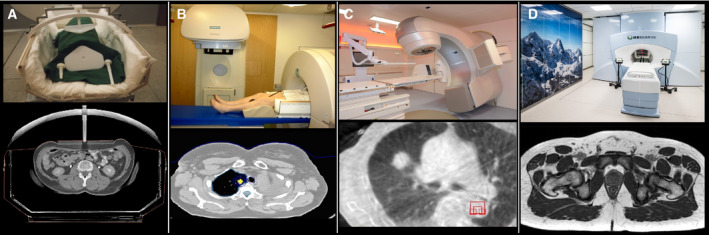
Development of image guidance in SBRT. (A) External stereotactic coordinates of the stereotactic body frame, (B) in‐room CT, (C) integrated CBCT, (D) integrated MRI imaging.

Ablating small targets with focal radiation has been practiced successfully since the 1950s for intracranial lesions, making radiosurgery (stereotactic radiosurgery) a noninvasive treatment option for functional and vascular disorders, benign and malignant tumors. However, multiple technological advances were required to transfer this concept from the neurocranium to targets located in the body: extracranial stereotactic RT, today called stereotactic body RT (SBRT), or stereotactic ablative RT (SABR), has been developed and first clinically introduced at the Karolinska Hospital in Sweden in 1994 [61] and shortly thereafter been pioneered by Japanese [62] and German [63,64] RT centers. SBRT was characterized by rigid patient positioning and immobilization in a stereotactic body frame, control of breathing‐induced target motion, conformal treatment planning by noncoplanar treatment techniques, inhomogeneous dose distributions in the target and dose delivery in few fractions of high single fraction doses Fig. 5.

Initial clinical experiences and early prospective trials of SBRT were made using conventional linacs with and the stereotactic body frame was the most relevant SBRT‐specific hardware innovation. This frame aimed to establish a system of external coordinates for locating the target volume at simulation and subsequently for targeting at the time of treatment. The replacement of this external stereotactic body frame by in‐room imaging has been the most relevant advancement of SBRT. In‐room imaging allows immediate visualization of the tumor before and during RT delivery through several approaches: stereoscopic X‐ray imaging; CBCT, and integrated MRI. Image guidance with online adjustment of the isocenter position, or online adaptive re‐optimization of the RT plan, improved the accuracy of SBRT through the compensation of interfractional and intrafractional variations in target and organ‐at‐risk position, shape, and volume, thereby minimizing unintentional exposure of normal tissue with ablative radiation doses. IGRT addresses both nonperiodical and periodical motion, in particular breathing‐induced motion in the thorax and upper abdomen. Further advances, among many others, enabled more accurate dose calculation and improved dose conformity with simultaneously rapid treatment delivery by dynamic intensity‐modulated RT.

It is important that these technological advances not only improved accuracy and therefore the safety and efficacy profile of SBRT, but also streamlined the process of SBRT planning and delivery. Improved accuracy combined with a reliable and efficient SBRT process was cornerstones for implementation of SBRT outside of specialized centers and outside of prospective clinical trials. Today, SBRT is therefore routinely practiced in the majority of RT centers [65]. In this section, the clinical evidence‐based rational for the use of SBRT will be summarized using two clinical examples: early‐stage NSCLC and OMD.

Traditionally, surgical lobectomy has been the only evidence‐based treatment option for early stage NSCLC offering a high probability of cure. RT has been indicated only in medically inoperable patients because treatment failure was observed in the majority of the patients. This is based on the observation that RT delivered in conventional fractionation with doses of about 60 Gy fails to achieve a high probability of local tumor control. Modeling has suggested that much higher radiation doses are necessary for eradication of NSCLC [66], and such doses are beyond normal tissue tolerance, if delivered to large volumes using conventional RT techniques. The accuracy of SBRT allowed for focal treatment with escalated and sufficiently high irradiation doses beyond 100 Gy biologically equivalent dose [67], which translated into long‐term local tumor control of 90% and improved overall survival. High rates of local tumor control combined with a favorable safety profile have been consistently demonstrated in prospective [68] and retrospective studies. Randomized controlled trials comparing conventionally fractionated RT with SBRT confirmed the superiority of SBRT [69,70] and population‐based studies demonstrated that implementation of SBRT allowed treatment of more patients with curative intent and thereby improved overall survival [71]. ESMO and NCCN guidelines therefore recommend SBRT as the treatment of choice for patients with inoperable stage I NSCLC (www.nccn.org, www.esmo.org). Initial results further indicate that SBRT may achieve similar or noninferior results [72] compared to surgical treatment of stage I NSCLC, however, prospective evidence is required to conform this hypothesis. Currently, clinical trials are addressing the value of biomarkers for early response assessment and the combination of SBRT with immune‐checkpoint inhibition to reduce the risk of regional and distant recurrences, despite the lack of phase I and phase II data, three large randomized phase 3 trials are currently addressing this question.

Oligometastatic disease has been defined as an intermediate state between early stage, where cure is the goal of radical local treatment, and systemic metastasized stage, where local and systemic therapy follows a palliative goal [73]. Although the term ‘oligometastases’ was coined and defined in 1995, surgical resection of solitary or limited metastases has been performed for decades and has achieved long‐term disease‐free survival and overall survival for selected patients. However, based on a systematic review of oligometastatic NSCLC, surgical resection was the exclusive local treatment modality until 2003 and the predominant modality until 2007 [74], with RT used in only very few patients. This is explained by the inability of conventional RT to locally eradicate oligometastases with sufficient safety and efficacy. Safety and efficacy of SBRT have been demonstrated in both primary and metastatic disease indicating that sufficiently high radiation doses can successfully sterilize metastases of histologies, which were previously assumed as radioresistant [75]. For pulmonary oligometastases of NSCLC, a matched pair analysis reported identical outcome of SBRT and surgical metastasectomy and similar promising results of SBRT have been described for other frequent oligometastases locations such as the liver, adrenal gland, bone metastases, and lymph node metastases [76]. The favorable therapeutic ratio combined with rapid adoption of SBRT was key to validate the concept of local ablative treatment for OMD in general. Until today, four randomized controlled trials evaluated the value of local ablative treatment of all macroscopic cancer sites in addition to standard of care systemic therapy: three randomized controlled trials reported improved overall survival in lung cancer [77], colorectal cancer [78], and in a disease agnostic setting [79],the fourth study was underpowered for OS but reported a significantly improved progression‐free survival [80]. Whereas radiofrequency ablation was the exclusive locally ablative treatment modality in the earliest CLOCC trial 17 [81], SBRT was the exclusive locally ablative treatment modality in two studies [79,80] and the most frequent in the study by Gomez *et al*. [77]. As a consequence, the current ESMO guideline for oligometastatic NSCLC states that the ‘relative contribution of surgery versus RT as local treatment modality has not been established yet’ in OMD (https://www.esmo.org/guidelines/lung‐and‐chest‐tumours).

**Fig. 6:**
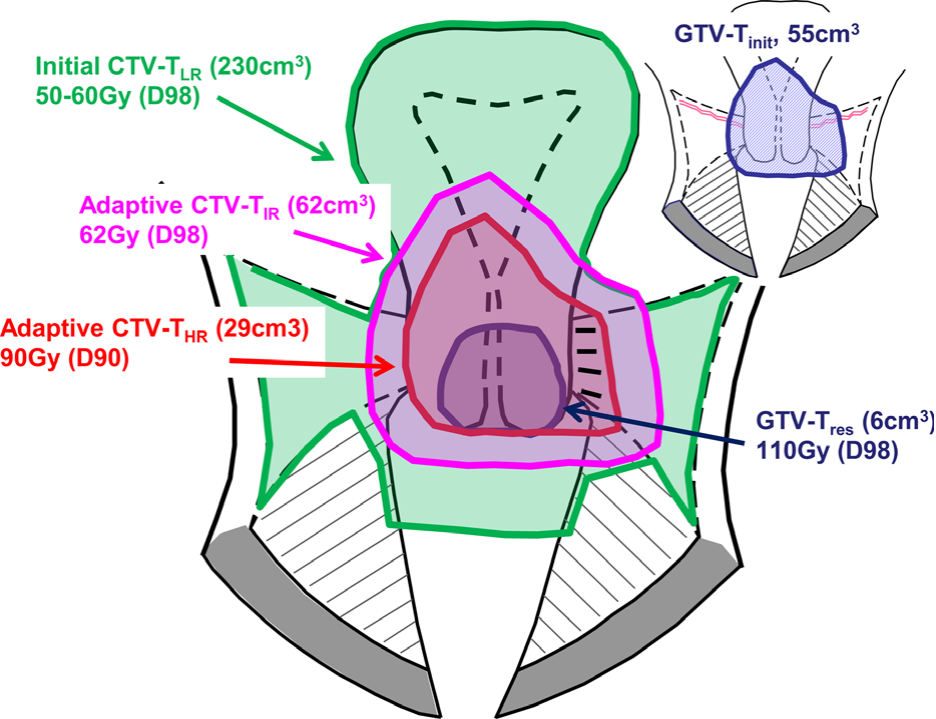
Median volumes and mean doses (D90 for adaptive CTV‐T_HR_, D98 for adaptive CTV‐T_IR_ and GTV‐T_res_) for cervix cancer (unpublished data from the EMBRACE I and II studies). In EMBRACE II, the median volume of GTV‐T_init_ is 55 cm^3^ (time of diagnosis). The extent of the GTV‐T_init_ is reflected in the adaptive CTV‐T_IR_ (time of brachytherapy), and this region received a median near‐minimum dose of 62 Gy in EMBRACE I. In good‐responding tumors, the dose at the border of the GTV‐T_init_ is 60–70 Gy, while in poor‐responding tumors, this region may receive doses similar to the adaptive CTV‐T_HR_ (e.g., around 80 Gy). Figure is modified from [88] Fig. 6.

Despite these progresses, there are many challenges which need to be addressed by future clinical and technological research Fig. 6. SBRT is constantly improved from a technical perspective to further improve its therapeutic ratio, especially for anatomical locations, such as the mediastinum or abdomen. Clinically, SBRT is being explored in the curative setting for example prostate and kidney cancer, as well as for palliative treatments, such as treatment of painful bony metastases [82, 83, 84]. Moreover, multimodal treatment concepts are developed to combine SBRT with modern targeted therapy and immunotherapy [85].

## 6 Image‐guided brachytherapy

Brachytherapy is a RT modality that uses sealed radioactive sources placed inside or in close proximity to a tumor target. The clinical precondition for brachytherapy applications is direct access to the tumor and a limited size tumor volume (up to 50–100 cm^3^). At present, the most common sites for administration of brachytherapy are gynecologic and prostate cancer. In image‐guided brachytherapy, the imaging is performed with the brachytherapy applicator in place, and it is possible to accurately depict the target in relation to the irradiator [86]. A stable relation between target and source of radiation makes it possible to treat accurately without the setup uncertainty margins needed in external beam RT. In general, brachytherapy treats a given target to higher doses, while involving less irradiation of normal tissue as compared to external beam RT [87].

For gynecological cancer, during the last two decades significant developments were achieved in brachytherapy through the integration of MRI for target definition [88]. The major conceptual innovation was to introduce MRI for identification of a response‐adaptive target concept. The ICRU89 report [89] and GEC ESTRO recommendations [90] have established a common terminology for target volumes with different risk of recurrence in locally advanced cervical cancer. Further GEC ESTRO recommendations follow a similar approach in primary vaginal cancer [91]. The adaptive approach includes target volumes defined at time of diagnosis and at the time of brachytherapy. Target volumes are defined at diagnosis:

- Initial GTV (GTV‐T_init_): the primary GTV.
- Initial high‐risk CTV (initial CTV‐T_HR_): the volume bearing the highest risk of recurrence; for cervical cancer, this includes the whole cervix as well as the GTV‐T_init_.
- Initial low‐risk target volume (initial CTV‐T_LR_): the compartments at risk of microscopic disease; for cervical cancer, this is uterus, parametria, (upper) vagina, and the anterior/posterior spaces toward bladder and rectum.

An adaptive CTV takes into account the morphology and topography of the GTV‐T_init_ as well as the response to treatment. For cervical cancer, the adaptive target volumes are defined at the time of brachytherapy. This is after delivery of external chemoradiation of 40–50 Gy, which is assumed sufficient to control microscopic disease. The adaptive target volumes are Fig. 6:

- Residual GTV (GTV‐T_res_): the residual gross tumor.
- Adaptive high‐risk CTV (adaptive CTV‐T_HR_): the volume bearing the highest risk for recurrence; for cervical cancer, this includes the GTV‐T_res_, the whole cervix, and adjacent residual pathologic tissue, if present.
- Adaptive intermediate‐risk CTV (adaptive CTV‐T_IR_): represents the GTV‐T_init_ as superimposed on the topography at the time of brachytherapy, together with a margin surrounding the adaptive CTV‐T_HR_.

In prostate cancer, the advent of trans‐rectal ultrasound (TRUS) and TRUS‐guided brachytherapy [92] had a profound and practice changing impact on the management of prostate cancer and expected cancer control outcomes [93] in patients with localized disease. This success was confirmed in subsequent randomized trials [9], but has also highlighted a pressing need to reduce the risk of urinary toxicity associated with prostate brachytherapy [94], including obstructive uropathy and pain.

One strategy currently under investigation is to integrate MRI and/or PET images in order to identify intraprostatic regions bearing dominant burden of cancer and considered at highest risk of recurrence [95]. The technical feasibility of such integration has been demonstrated through both computational (MRI/TRUS fusion) [96] and MRI‐only methods [97], but a clinical impact on patient outcomes remains to be demonstrated.

In the following, the impact of image guidance on treatment approaches in different types of diseases is discussed. In cervix cancer, the introduction of image guidance and response‐adaptive target volumes had major impact on the treatment approach [98]. Image‐guided brachytherapy takes into account both status at diagnosis and treatment response, and the approach has become highly individualized. The variable risk of recurrence in the different target volumes is taken into account through risk adaptive dose prescription. Typical total external beam and brachytherapy dose administration in cervix cancer is (EQD2 doses): 45–50 Gy to regions with suspected microscopic spread at diagnosis (initial CTV‐T_LR_), > 60 Gy to regions with suspected microscopic spread defined at time of brachytherapy (D98, adaptive CTV‐T_IR_), > 85 Gy to regions with major risk of (residual) macroscopic disease at brachytherapy (D90, adaptive CTV‐T_HR_), > 90 Gy to regions with residual GTV at brachytherapy (D98, GTV‐T_res_) Fig. 6. The remarkable variation in dose prescription across the different target volumes is facilitated by the high brachytherapy dose gradient. Image guidance and adaptation of application technique (addition of interstitial needles) in cervix cancer have considerably improved the target dose coverage while the overall irradiated volumes have been significantly reduced [99]. The change of practice has involved dose escalation in patients with advanced disease and poor response to external beam RT (e.g., through the use of interstitial needles [100], and dose de‐escalation in patients with limited disease and/or favorable response [101].

In prostate cancer, the integration of images that depict tumor within the prostate can facilitate a dose‐painted approach, whereby treatment can be de‐intensified in low‐risk regions in order to reduce toxicity while maintaining cancer control. Although still considered investigational, this strategy has gained momentum and has been integrated in a prospective phase III randomized clinical trial (NCT02960087). At its extreme, this strategy also leads to the concept of focal brachytherapy, which may be appropriate in select patient subgroups and contexts.

The tolerance of tissue to the highly potent target dose prescription (e.g., > 85–90 Gy) in both gynecological and prostate cancer brachytherapy is likely explained by these considerable dose levels being applied to only limited volumes. For example, in cervix cancer, median volumes for GTV‐T_res_ (median D98 of 110 Gy) and adaptive CTV‐T_HR_ (median D90 of 90 Gy) correspond to 6 cm^3^ and 29 cm^3^, respectively Fig. 6.

The clinical evidence for improved outcome (disease control and less morbidity) of image‐guided brachytherapy in cervix cancer has been demonstrated through mono‐institutional reports and the large EMBRACE multicenter studies: retroEMBRACE, EMBRACE I, and EMBRACE II [102]. Retro‐EMBRACE demonstrated excellent local and pelvic control with 3‐year actuarial pelvic control of 96%, 89%, and 73% in stage IB, IIB, and IIIB disease, respectively [103]. This is superior to reports of chemoradiation [104,105] with an overall increase in pelvic control of around 10%. The overall survival was similar to results from randomized chemoradiation trials [106] but around 12% better than large population‐based cohorts treated with chemoradiation [105,107]. At the same time, major morbidity was limited after image‐guided adaptive RT (3–6% per organ) [103,108, 109, 110, 111].

In prostate brachytherapy, although clinical evidence of improved disease control with prostate gland targeting under TRUS guidance is strong, high‐level evidence of decreased morbidity with an MRI‐guided and tumor‐targeted approach is lacking. Ongoing randomized trials of focal brachytherapy boost to external beam RT (NCT04100174), or dose‐painted brachytherapy (NCT02960087) are expected to report in the coming years.

In conclusion, the principles of image‐guided and response‐adaptive image‐guided brachytherapy have the potential to improve outcomes in other cancers, where definitive RT is delivered as a combination of external beam RT, followed by a brachytherapy boost. Such cancers include rectum, anal canal, breast, head and neck, esophagus, and lung. Image guidance for brachytherapy alone is applied also in prostate, liver, and eye tumors.

## 7 Quantitative imaging for response‐adaptive radiation oncology

**Fig. 7:**
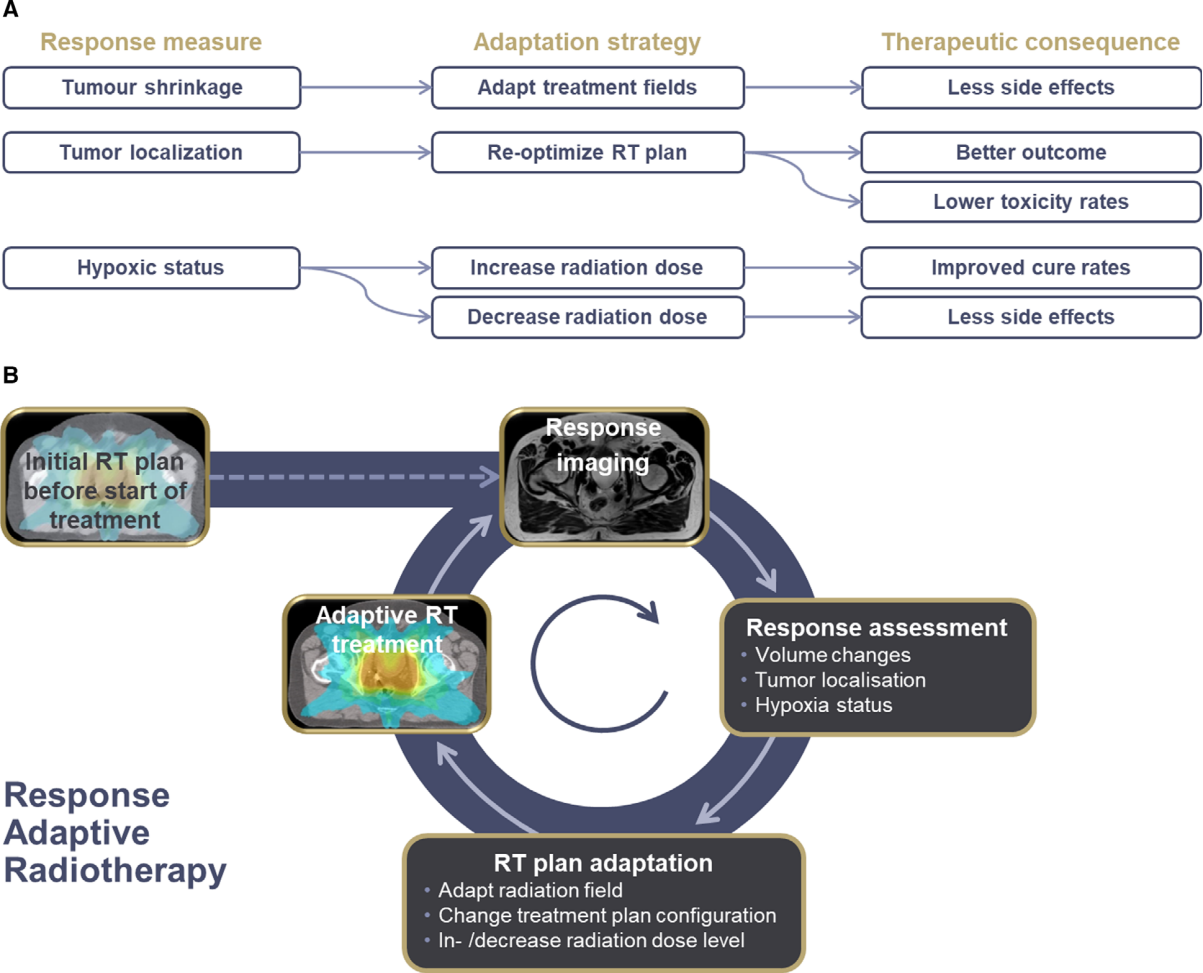
(A) Schematic representation of different putative adaptation measures, subsequent adaptation measures, and resulting therapeutic consequences. (B) Illustration of a ART workflow: Before the start of treatment, an optimal treatment plan is generated. During treatment, systematic feedback measurements about tumor response are taken into account in order to adapt RT in terms of field size, target, or radiation dose level to yield most optimal therapeutic results.

Increasing availability of modern functional imaging techniques, such as MRI or PET, allows for precise anatomical, functional, and biological characterization of tumors before and during RT treatments. With these modern imaging techniques, it is possible to assess individual response to RT already in an early phase of treatment, several weeks before the end of therapy. Modern RT equipment permits individually modifying the treatment according to the information about individual response Fig. 7A. Consequently, response‐adaptive RT (ART) is defined as ‘a radiation treatment process where the treatment plan can be modified using a systematic feedback of measurements (treatment position variation due to beam displacement and target geometric variation)’ [112]. Information about response to RT, such as tumor volume reduction or characteristic changes in functional or biological markers of tumor aggressiveness, can be optimally assessed using modern imaging techniques [113]. Triggered by individual response measures, image‐guided adaptive RT provides the technical basis to achieve optimal cure rates for patients and at the same time keep the risk for treatment‐related side effects as low as possible.

Clinical trials involving large numbers of patients suffering from cervical cancer have shown the effectiveness of adaptive RT taking into account tumor shrinkage at the end of initial chemoradiation, with a risk‐adapted radiation dose prescription to different tumor regions, and adaptation of the treatment technique according to individual response and adjacent OAR [88]. Further evidence of improved treatment results has been generated by large controlled trials on adaptive RT in NSCLC patients [114]. The results provided by these studies demonstrated a high clinical impact of ART as adjusting the radiation treatment plan according to changes in the anatomical situation guarantees maintained or even increased radiation dose levels in the tumor region while reducing treatment‐related toxicities [114, 115, 116]. Also in head‐and‐neck cancer patients, several smaller trials have proven the benefit of ART in terms of increasing the therapeutic ratio by increasing the radiation dose to the tumor in a calculable way and therefore sparing adjacent normal tissues [117].

Recent clinical trials have shown not only that changes in the anatomical situation observed during treatment should be considered during RT, but also that changes in functional and biological tumor characteristics need to be taken into account for ART. Several studies have shown that information provided by PET imaging and functional MRI reveal information about the individual aggressiveness of individual tumors and are prognostic for RT outcome [118, 119, 120, 121]. Moreover, clinical trials have shown that characteristic changes in tumor hypoxia assessed with PET during the first 2 weeks of RT are associated with treatment outcome [122,123]. Thus, ART taking into account functional information obtained during image guidance can help overcome biology‐induced radiation resistance by increasing the radiation dose at resistant regions inside the tumor (dose painting, Fig. 7B. A similar concept is investigated in a currently running trial in head‐and‐neck cancer patients where radiation dose to lymph node metastases is de‐escalated upon early response in PET imaging. This might result in less toxicity without jeopardizing safety [49].

While there is an increasing number of studies investigating the association between functional imaging biomarkers and individual response to RT [44,119, 120, 121, 122,124, 125, 126], there is a wide variability in the methodology of acquisition and data analysis with respect to functional imaging. To improve consistency between studies, quantitative imaging techniques need to be applied for the assessment of functional tumor characteristics. Quantitative imaging is defined as the extraction of quantifiable measures from medical images for the assessment of physiological quantities with respect to tumor or tissue characterization. As functional imaging data have a direct impact on the radiation dose used to treat the patient, quantitative imaging techniques are a major prerequisite for image‐guided radiation oncology [127].

Current RT techniques, using, for example, the MRI‐Linac, allow for highest geometric precision due to real‐time image guidance [37,42,45,128, 129, 130, 131].

In addition, with the clinical availability of online MRI‐guided RT as described above, it is possible to acquire quantitative MRI data at the time of RT treatment and adapt the treatment according to functional response measures in real‐time [132]. Consequently, quantitative imaging‐based ART is a modern, high‐precision RT solution, which empowers better cancer cure rates and at the same time reduces the risk for side effects.

## 8 The challenge of IGRT technology assessment

Image‐guided radiotherapy is driven by biological concepts, clinical need, and technology developments. The combination of these three main factors in addition to the short innovation cycles results in a major challenge for radiation oncology. First of all as in all other areas of radiation oncology, before a new type of IGRT is deployed in patients, a rigorous testing and evaluation are mandatory to ensure patient safety. A new biological concept such as dose painting, where imaging is the enabler to change the dose distribution within tumors, needs prospective clinical trials including ultimately a comparative randomized phase 3 trial. A new IGRT technology that is transforming radiation oncology such as MR‐linacs requires a comprehensive framework as described in the R‐IDEAL concept [36]. Stepwise clinical trials toward comparative randomized trials are the gold standard of evidence‐based medicine. The conduct of these trials in the context of IGRT is challenged by the high costs, conceptual controversies in the design of health technology assessment trials (e.g., [133], and the contrast between short innovation cycles and long‐term clinical endpoints. The latter may make data obsolete at the time they become available to the public because clinical and technological standards have been changed during the follow‐up period of a trial. In addition, not all patients even with the same type of cancer will benefit from new IGRT technology. To overcome this problem, model‐based approaches have been introduced in the assessment of proton therapy in head and neck cancer [134]. It is generally accepted that prospective observational studies are sufficient to validate more incremental steps in the application of existing IGRT technology. Examples include IGRT [135] and ART [114] for lung cancer. However, for most of the innovations in IGRT clinical trials including phase 3 trials are essential to demonstrate cost‐benefit of the new technology. To address the above‐mentioned challenges, these clinical trials have to be performed in academic European and international networks which need substantial support by funding agencies, universities, and industry. Only a joint effort of the leading academic centers will ensure that new technology can be offered timely for the benefit of every cancer patient without jeopardizing patient safety or violating the principles of evidence‐based medicine as well as solidarity in healthcare systems.

## 9 Summary

High‐resolution IGRT has become a mainstay of modern RT. In‐room medical imaging with portal imaging, CT, MR, or ultrasound has changed clinical practice, that is, providing the prerequisite to safely deliver radiation dose. The proven concept that IGRT enables full tumor coverage with sufficient radiation dose and sparing normal tissue allows improving the outcome of many cancer patients belongs to the major contributions of radiation oncology in cancer medicine. IGRT will remain a driving force for research and development, for example, to create novel types of radiation treatments. IGRT as a core technology will facilitate the current shift of paradigm toward personalized radiation oncology: imaging biomarkers for repeated, noninvasive, point‐of‐care multiscale molecular tissue profiling which will guide individualized dose prescription and real‐time response adaptation embedded in automatized workflows and digitalized environments. With its track records and future perspectives, image‐guided radiation oncology will be a cornerstone toward better cures in the future for all cancer patients.

### Box

#### Glossary

- CBCT (cone‐beam computed tomography): a special form of computed tomography which can be installed on a linear accelerator to enable IGRT.
- Dose painting: a concept in radiation oncology which proposes to deliver nonhomogeneous radiation doses according to the radiation sensitivity and treatment response of parts of the tumor. Conventional radiotherapy delivers the same dose to the entire tumor. In dose painting imaging for example with PET is used to visualize parts of the tumor which are more resistant than others. These parts are then treated with higher radiation doses.
- Dual‐energy CT: a special form of computed tomography that uses different sources and energies.
- Volumetric‐modulated arc therapy (VMAT): Volumetric arc therapy is a special form of highly conformal intensity‐modulated radiotherapy (IMRT) where the rotating treatment beam is continuously adapted to cover the tumor and to spare the normal tissues.
- Stereotactic ablative radiotherapy (SABR): A treatment concept where a single or few fractions with large radiation doses intended to ablate all cancer cells are delivered with high precision using IGRT. Often used in small tumors or limited metastases.
- Brachytherapy: A special form of radiotherapy where a radiations source is permanently or temporarily positioned directly or close to the tumor.
- Gross tumor volume (GTV): tumor visible to the naked eye or visualized with medical imaging.
- Clinical target volume (CTV): space surrounding the GTV which might contain cancer cells which have infiltrated adjacent tissues. These cancer cells are invisible by naked eye or medical imaging. Radiation therapy with curative intent needs to cover this space in order to inactivate all cancer cells.
- Planning treatment volume (PTV): takes uncertainties in the precision of treatment delivery, for example, motion of the organs during treatment delivery or uncertainties in patient positioning, into account. PTV represents a composite of GTV and CTV plus a safety margin. Radiation oncologists prescribe the radiation dose typically to the PTV.
- Oligometastatic disease (OMD): A concept that proposes a state of limited metastases in the course of the cancer disease where local therapies such as SABR might be used.
- Positron Emission Tomography (PET): is a medical imaging modality visualizing metabolism (e.g., glucose metabolism) or pathophysiological process (e.g., tumor hypoxia).
- Linear accelerator (Linac): most often used treatment device for external beam radiotherapy.
- Adaptive radiotherapy (ART): a concept in radiation oncology which takes the changes of the tumor during beam delivery (e.g., motion) and during the course of fractionated radiotherapy (e.g., tumor shrinkage) into account by adapting the treatment for example through smaller PTV or through individualized radiation dose according to response.

## Conflict of interest

DZ and DT receive financial and technical support from Elekta AB (Stockholm, Sweden) under a research agreement. For the MRgRT program in Tübingen, DZ and DT receive funding by the German Research Council (PAK 997/1, ZI 736/2‐1), University Hospital Tübingen, and Medical Faculty Tübingen. DZ and DT receive sponsoring for travels and scientific symposia from Elekta, Siemens, Philips, and Dr. Sennewald. DZ and DT confirm that none of the above‐mentioned funding sources were involved in the study design, in the collection, analysis, and interpretation of data and in the writing of the paper. The department of Radiation Oncology at Amsterdam UMC received research support from Varian medical systems and Viewray Inc.

## Author contributions

The chapters were written by the following authors: DZ helped in writing of introduction, summary and the chapter on challenge of IGRT technology assessment. MvH performed CT‐based IGRT. BJS conducted the 0.35T hybrid MR‐linac. JJWL conducted high‐field MR‐linac. VG and KH performed molecular imaging with positron emission tomography in radiation oncology. MG involved in image guidance in high‐precision stereotactic ablative radiation oncology. KT, CM, and RP performed image‐guided brachytherapy in radiation oncology. DT involved in quantitative imaging for response‐adaptive radiation oncology.
